# Impacts of natural factors and farming practices on greenhouse gas emissions in the North China Plain: A meta‐analysis

**DOI:** 10.1002/ece3.3211

**Published:** 2017-07-21

**Authors:** Cong Xu, Xiao Han, Roland Bol, Pete Smith, Wenliang Wu, Fanqiao Meng

**Affiliations:** ^1^ Beijing Key Laboratory of Biodiversity and Organic Farming College of Resources and Environmental Sciences China Agricultural University Beijing China; ^2^ Institute of Bio‐ and Geosciences, Agrosphere (IBG‐3) Forschungszentrum Jülich GmbH Jülich Germany; ^3^ Institute of Biological and Environmental Sciences University of Aberdeen Aberdeen UK

**Keywords:** farming practice, fertilizer, meta‐analysis, methane, natural factor, nitrous oxide

## Abstract

Requirements for mitigation of the continued increase in greenhouse gas (GHG) emissions are much needed for the North China Plain (NCP). We conducted a meta‐analysis of 76 published studies of 24 sites in the NCP to examine the effects of natural conditions and farming practices on GHG emissions in that region. We found that N_2_O was the main component of the area‐scaled total GHG balance, and the CH_4_ contribution was <5%. Precipitation, temperature, soil pH, and texture had no significant impacts on annual GHG emissions, because of limited variation of these factors in the NCP. The N_2_O emissions increased exponentially with mineral fertilizer N application rate, with *y *=* *0.2389e^0.0058*x*^ for wheat season and *y *=* *0.365e^0.0071*x*^ for maize season. Emission factors were estimated at 0.37% for wheat and 0.90% for maize at conventional fertilizer N application rates. The agronomic optimal N rates (241 and 185 kg N ha^−1^ for wheat and maize, respectively) exhibited great potential for reducing N_2_O emissions, by 0.39 (29%) and 1.71 (56%) kg N_2_O‐N ha^−1^ season^−1^ for the wheat and maize seasons, respectively. Mixed application of organic manure with reduced mineral fertilizer N could reduce annual N_2_O emissions by 16% relative to mineral N application alone while maintaining a high crop yield. Compared with conventional tillage, no‐tillage significantly reduced N_2_O emissions by ~30% in the wheat season, whereas it increased those emissions by ~10% in the maize season. This may have resulted from the lower soil temperature in winter and increased soil moisture in summer under no‐tillage practice. Straw incorporation significantly increased annual N_2_O emissions, by 26% relative to straw removal. Our analysis indicates that these farming practices could be further tested to mitigate GHG emission and maintain high crop yields in the NCP.

## 1 INTRODUCTION

Global atmospheric concentrations of greenhouse gases (GHGs) such as CO_2_, N_2_O, and CH_4_ have continued to increase, which has further heightened public and scientific concerns (IPCC, 2014; Wei, Zhang, Chen, Zhang, & Zhang, 2012). N_2_O and CH_4_, mainly derived from the agricultural sector (Smith et al., 2007), have 265 and 28 times greater global warming potentials than CO_2_ over a time horizon of 100 years (IPCC, 2014). Although a number of climate change mitigation measures have been adopted in China during recent years, requirements for further mitigation of the continued increase in GHG emission are still much needed (Chen et al., 2014).

The North China Plain (NCP) occupies 23% of national cropland area (Ding, Cai, Cai, Yagi, & Zheng, 2007) and accounts for 43% of total winter wheat (*Triticum aestivum* L.) and summer maize (*Zea mays* L.) production in China (Shi et al., 2013). High land productivity in the NCP has relied on intensive farming practices since the 1980s (Liao, Wu, Meng, Smith, & Lal, 2015), which are characterized by frequent irrigation (Wang, Yu, Wu, & Xia, 2008) and high levels of mineral nitrogen (N) fertilizer application (550–600 kg N ha^−1^ year^−1^; Ju et al., 2009). However, in the near future, greater crop yields with reduced GHG emissions must be achieved in China to meet the dual goals of ensuring food security and reducing negative environmental impacts (Chen et al., 2014; The State Council of China, 2016).

Agricultural practices regulate soil N and carbon (C) dynamics and thereby affect the fluxes of N_2_O and CH_4_ (Adviento‐Borbe, Haddix, Binder, Walters, & Dobermann, 2007; Mutegi, Munkholm, Petersen, Hansen, & Petersen, 2010). Natural factors also affect or interact with farming practices, thereby influence N_2_O and CH_4_ emissions (Chatskikh, Olesen, Berntsen, Regina, & Yamulki, 2005; Čuhel et al., 2010; Gu et al., 2013; Jansen, 2009; Smith, 1997; Vidon, Marchese, Welsh, & Mcmillan, 2016). In recent decades, many site‐specific studies have been conducted to explore the impacts of fertilization (Tan et al., 2017; Yan, Yao, Zheng, & Liu, 2015), tillage (Tian et al., 2012; Wei et al., 2012), and crop residues (Hu et al., 2013; Huang, Gao, Christie, & Ju, 2013) on GHG emission and crop yield in the NCP. However, these individual studies were not able to provide a generalized understanding across this large region. Therefore, a comprehensive quantitative analysis of published studies regarding the NCP is necessary to assess the overall relationship between GHG emissions and natural and farming factors. Meta‐analysis was selected for this purpose, because it is a powerful method to integrate site‐specific results and draw overall conclusions at regional and global scales (Gurevitch, Curtis, & Jones, 2001; Luo, Wang, & Sun, 2010).

Previous meta‐analyses for China's agricultural soils have examined the relationship between natural and farming factors and GHG emissions (Lu, Huang, Zou, & Zheng, 2006; Zhao et al., 2016). However, they did not focus on winter wheat–summer maize (WW‐SM) rotation, the typical and major farming system in the NCP, and their conclusions did not provide technical support for GHG mitigation in the region. In this study, we calculated both response ratios and average amounts of GHG emission under different natural factors and farming practices. Regression analysis has also been used to obtain relationships between N_2_O emissions, emission factors (EFs, percentage of fertilizer‐induced N_2_O emission), crop yields, and N application rates. We aimed at quantifying the comprehensive responses of GHG emissions to major farming practices and natural factors in the NCP, which will facilitate large crop yields and GHG mitigation in the region.

## 2 MATERIALS AND METHODS

### 2.1 Data collection

We conducted a literature survey of peer‐reviewed papers published prior to April 2016 and collected data on N_2_O/CH_4_ emissions, climate and soil factors, farming practices, and crop yields for WW‐SM systems in the NCP region. All the papers were obtained from the databases of China National Knowledge Infrastructure (CNKI, the largest Chinese academic journal database) and Web of Science. We conducted a preliminary search using the keywords “N_2_O,” “CH_4,_” and “NCP.” We then selected papers based on the following selection criteria: (1) Studies must have been of the NCP under WW‐SM cropping systems; (2) measurements of N_2_O and/or CH_4_ fluxes must have been made under field conditions in the entire growth period of the wheat and/or maize cropping season, using static chamber methods; (3) cumulative GHG fluxes during the entire season, measurement frequency, and the number of field replications had to be reported. By applying these selection criteria, 76 papers were selected for study (56 for N fertilization, 19 for tillage, 29 for straw management, 13 for slow‐release fertilizer (SRF) application, and 24 for organic fertilizer application; Appendix S1). Some authors published their results on grain yield and GHG emission separately in different papers, so in some cases missing yield data were collated from different publications by the same authors. For each study, the GHG emission or crop yield for each individual treatment combination was separated as distinct single data points in our meta‐analysis. Unless available in the original literature, precipitation and temperature during the experimental period of each study were obtained from the China Meteorological Data Service Center (http://data.cma.cn). To avoid bias toward multiyear studies, the mean value of measurements in different years was used as a single observation when experiments were repeated over time, except for analysis of the effects of weather conditions (precipitation and temperature).

### 2.2 Data analysis

#### 2.2.1 Calculation of total GHG balance

We used the IPCC coefficients to calculate CO_2_‐equivalents (CO_2_‐eq) of N_2_O and CH_4_ emissions over a 100‐year time horizon (298 and 25 for N_2_O and CH_4_, respectively; IPCC, 2007). The overall CO_2_‐eq of N_2_O and CH_4_ emission was expressed as total GHG balance (Cherubini, 2010). Area‐scaled and yield‐scaled data represented the total GHG balance per unit crop field (ha) and per unit crop yield (Mg), respectively. The equations are as the follows.

(1) $$\text{Area‐scaled total GHG balance}\;=\;\frac{N_{2}O\;\times \;44}{28}\;\times \;298+\frac{{\text{CH}}_{4}\times 16}{12}\;\times \;25$$

(2) $$\text{Yield‐scaled total GHG balance}\;=\;\frac{\text{area‐scaled total GHG balance}}{\text{yield}}$$

Equations (1) and (2) were used to calculate area‐scaled (kg CO_2_‐eq ha^−1^ season^−1^ or year^−1^) and yield‐scaled (kg CO_2_‐eq Mg^−1^ season^−1^ or year ^−1^) GHG balance, respectively, where N_2_O is the N_2_O emission (kg N_2_O‐N ha^−1^ season^−1^ or year ^−1^), CH_4_ is the CH_4_ emission (kg CH_4_‐C ha^−1^ season^−1^ or year ^−1^), and yield is the crop yield (Mg ha^−1^ season^−1^ or year ^−1^).

#### 2.2.2 Natural factors

CO_2_‐equivalent N_2_O and CH_4_ emissions for fertilization levels of ≥200 kg N ha^−1^ season^−1^ or ≥400 kg N ha^−1^ year ^−1^ from each study were selected to evaluate the impacts of soil pH and soil texture on GHG emissions. Soil pH was divided into two levels (6.5–7.5 and >7.5), which represent neutral and alkaline soils, respectively. Soil textures in the meta‐analysis were categorized according to the USDA classification system. To avoid limiting the number of samples in each texture class, we classified the textures by clay content into two types, sandy loam and loam to clay loam. We used the methods of Linquist, Van Groenigen, Adviento‐Borbe, Pittelkow, and Van Kessel (2012) to conduct the meta‐analysis, and the equations used were as follows.

(3) $$M=\frac{\Sigma \left(Y_{i}\times W_{i}\right)}{\Sigma W_{i}}$$

(4) $$W_{i}=\frac{n\times f}{\text{obs}}$$

Equation (3) was used to calculate weighted mean values of GHG emissions or area‐scaled total GHG balance under different natural conditions, in which *Y*_*i*_ is the observation of GHG emission or total GHG balance at the *i*th site, *M* is the mean value of CO_2_‐eq GHG emission or area‐scaled total GHG balance (kg CO_2_‐eq ha^−1^ season^−1^ or year ^−1^), and *W*_*i*_ is the weight for observations at the *i*th site, which was calculated using Equation (4). In that equation, *n* is the number of replicates in the field experiment, *f* is the number of GHG measurements per month, and obs is the total number of observations at the *i*th site. To prevent studies with high sampling frequencies from being assigned extreme weights, a maximum value *f *=* *5 was assigned when GHG fluxes were measured more than once per week. Linear regression was used to examine the relationship of N_2_O emissions with precipitation and temperature during the experimental period.

#### 2.2.3 Farming practices

Response ratio (*R*) was used to evaluate the impacts of farming practices on N_2_O emissions, CH_4_ emissions, crop yield, and total GHG balance (area‐scaled and/or yield‐scaled). Only studies that included side‐by‐side comparisons were selected for this calculation. The rates of applied N were separated into three levels (50–150, 150–250, and 250–350 kg N ha^−1^ season^−1^ or 100–300, 300–500, and 500–700 kg N ha^−1^ year ^−1^). N fertilizers in the selected studies were mainly ammonium‐based (e.g., urea) in the study region (Ju et al., 2009). In addition to the N application rate, five types of fertilization measures in NCP were assessed: mineral fertilizer application alone (M), full‐dose mineral fertilizer plus organic manure (M+O), reduced mineral fertilizer combined with organic manure (RM+O, with a total N dose equivalent to M treatment), application of organic manure alone (O) and application of SRF. We divided the tillage measures into no‐tillage (NT) and conventional tillage (CT), and straw management into straw incorporation and straw removal. To evaluate the effect of straw incorporation under N fertilization, the effects of straw incorporation on N_2_O emission were further separated into with and without N fertilizer application. CH_4_ emissions were all found to be negative in the side‐by‐side comparisons. We used CH_4_ uptake in the calculation of response ratios to avoid confusion when understanding effect sizes.

The natural log of the response ratio (ln*R*) was calculated as an index of the effect size:

(5) $$\ln R=\;\ln\;\frac{X_{t}}{X_{c}}$$

where *X*_*t*_ and *X*_*c*_ are measurements of N_2_O emission, CH_4_ uptake, yield, or total GHG balance (area‐scaled and/or yield‐scaled) for the treatment and control (Table 1), respectively. The mean of the response ratios ($\overset{¯}{R}$) was calculated from ln*R* values of individual studies using Equation (6):

(6) $$\overset{¯}{R}=\;\text{exp}\;\frac{\sum (\ln R_{i}\times W_{i})}{\sum W_{i}}$$

where *W*_*i*_ is the weighting factor, estimated by Equation (4). To facilitate interpretation, results of the *R* analysis were reported as percentage change under the treatment relative to the control $\left([\overset{¯}{R}-1]\times 100\right)$.

**Table 1:** Treatments and corresponding controls in the calculation of response ratio

Management	Treatment	Control
N application	N application rates under various intervals	No N fertilization
Tillage	NT	CT
Straw	Straw incorporation	Straw removal
Organic manure	M+O, RM+O, and O	M
Slow‐release fertilizer	SRF	M

In addition to the calculation of *R*, we calculated absolute values of mean GHG emission and area‐scaled total GHG balance under different levels of N application or farming practice. Mean values were then evaluated using the same approach as described in Section 2.2.2, with *M* in Equation (3) representing the mean value of N_2_O emissions (kg N_2_O‐N ha^−1^ season^−1^ or year ^−1^), CH_4_ emissions (kg CH_4_‐C ha^−1^ season^−1^ or year ^−1^), or area‐scaled total GHG balances (Mg CO_2_‐eq ha^−1^ season^−1^ or year ^−1^) under various treatments.

#### 2.2.4 Statistical and regression analysis

All studies that reported either N_2_O emission or crop yield were included to determine best‐fit regression curve models for N_2_O emission or yield as functions of the N application rate. Linear, exponential, quadratic, and linear‐plateau models (Cerrato & Blackmer, 1990) were tested with each dataset. We used the Statistical Analysis System (SAS Institute, 1998) package for statistical analyses and evaluation of significance levels. If statistical significance was detected for several models at the critical level of 5%, we then selected the model with the largest coefficients of determination (*R* ^2^). The relationships between N application rate and EF of N_2_O were subsequently generated, based on the above best‐fit regression curves for N_2_O emissions in response to the N application rate.

#### 2.2.5 Meta‐analysis

The meta‐analysis was performed using MetaWin 2.1 (Rosenberg, Adams, & Gurevitch, 2000). A random‐effect model was used to calculate the mean effect size. We used bootstrapping (4,999 iterations) to generate these mean emissions, total GHG balances, effect sizes, *p*‐values, and 95% bootstrapped confidence intervals (95% CIs). Mean effect sizes were only considered significantly different if their 95% CIs did not overlap. Sensitivity analysis was conducted for absolute values and response ratios to test whether the weighted and unweighted approach give similar results. The results using the weighted approach were very similar to that using unweighted approach, hence we only report the results of the former approach herein.

## 3 RESULTS

### 3.1 Natural factors

**Figure 1:**
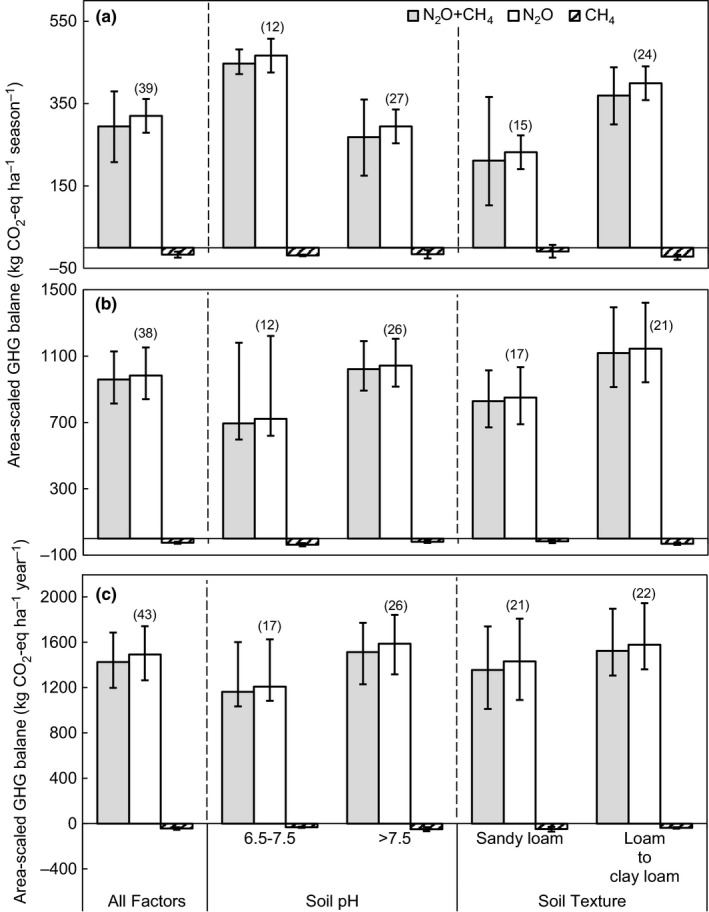
Area‐scaled GHG balance of N_2_O, CH_4,_ and N_2_O+CH_4_ under conventional fertilization for (a) wheat season, (b) maize season, and (c) annual period, which are categorized into different levels/types of soil pH, soil texture, and all factors. Figures in parentheses indicate number of observations. All error bars represent 95% confidence intervals

When all observations were taken into account, average N_2_O emissions during the wheat season, maize season, and annual period were 320 (232–400, 95% CI), 983 (841–1,153, 95% CI) and 1,492 (1,264–1,742, 95% CI) kg CO_2_‐eq kg ha^−1^, respectively (Figure 1a–c). This indicates significantly higher N_2_O emissions in the maize season (about three times that of the wheat season; *p < *.05). Average CH_4_ emissions were all found to be negative, suggesting that the agricultural soils of the NCP act as an overall sink for atmospheric CH_4_. When expressed as CO_2_‐eq, the CH_4_ uptake was much less than N_2_O emission, that is., <5% of the area‐scaled total GHG balance, indicating that the overall area‐scaled total GHG balance was predominantly determined by N_2_O emission. Therefore, we mainly address the trends of N_2_O emission in this section.

The N_2_O emission tended to be higher in loam to clay loam textured soils than in sandy loam soils, but a significant difference between these two soil textures was only detected for the wheat season (Figure 1a; *p < *.05). No pronounced differences in CH_4_ uptake or area‐scaled total GHG balance were found between soil texture categories (*p > *.05).

In the wheat season, N_2_O emissions and area‐scaled GHG balances in soils with pH of 6.5–7.5 were significantly greater than those with pH > 7.5 (*p *<* *.05; Figure 1a), but pronounced differences were not found for maize season and at annual scale (*p *>* *.05; Figure 1b,c). Across all periods, no statistical differences of CH_4_ emission were detected between neutral (pH 6.5–7.5) and alkaline (pH > 7.5) soils (*p ˃ *.05; Figure 1a–c).

**Figure 2:**
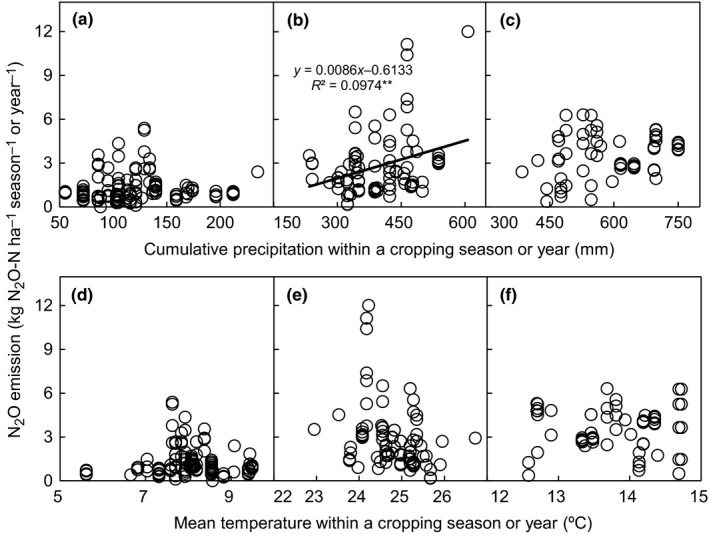
N_2_O emissions versus cumulative precipitation for (a) wheat season, (b) maize season, and (c) annual period, and N_2_O emissions versus mean temperature for (d) wheat season, (e) maize season, and (f) annual period. ** represents .01 significance level

N_2_O emission significantly increased with precipitation in the maize season (*p *<* *.01; Figure 2b), but there was no apparent relationship between the two in the wheat season and annual period (Figure 2a,c). The N_2_O emission also showed no significant relationship with temperature (Figure 2d–f).

### 3.2 N application rate

N_2_O emissions under the lowest N application rate (50–150 kg N ha^−1^ season^−1^ or 100–300 kg N ha^−1^ year^−1^) were 0.57, 0.51, and 1.37 kg N_2_O‐N ha^−1^ for the wheat season, maize season, and annual period, respectively. The N_2_O emissions increased dramatically to 1.14, 2.24, and 3.86 kg N_2_O‐N ha^−1^, respectively, under the highest N application rate (250–350 kg N ha^−1^ season^−1^ or 500–700 kg N ha^−1^ year^−1^; *p < *.05; Table 2). The area‐scaled total GHG balance showed trends similar to N_2_O emission, which increased from 0.60 CO_2_‐eq ha^−1 ^year ^−1^ under the lowest N application rate to 1.75 CO_2_‐eq ha^−1^ year ^−1^ for the highest rate (Table 2). N application rates also had a significant effect on the absolute amount of CH_4_ uptake in the maize season (*p < *.01; Table 2).

**Table 2:** N_2_O emission, CH_4_ emission, and area‐scaled total GHG balance for wheat season, maize season, and annual period, as affected by N application rate

	N rate	Mean N rate	Obs^a^	N_2_O emission	95% CI	*p*	CH_4_ emission	95% CI	*p*	Area‐scaled total GHG balance	95% CI	*p*
	kg N ha^−1^			kg N_2_O‐N ha^−1^			kg CH_4_‐C ha^−1^			Mg CO_2_‐eq ha^−1^		
Wheat	0	0.0	5	0.37	0.23~0.59	<.01	−1.02	−1.61 to −0.66	.70	0.14	0.08~0.23	<.01
	50–150	117.0	7	0.57	0.3~0.87		−0.62	−0.97 to −0.17		0.25	0.13~0.37	
	150–250	216.7	23	0.84	0.66~1.11		−0.73	−0.99 to −0.56		0.35	0.24~0.46	
	250–350	311.6	14	1.14	1.00~1.35		−0.73	−0.92 to −0.57		0.51	0.44~0.61	
Maize	0	0.0	7	0.47	0.42~0.59	<.01	−0.51	−0.8 to 0.33	<.01	0.20	0.18~0.26	<.01
	50–150	100.3	10	0.51	0.40~0.71		−0.26	−0.43 to −0.09		0.23	0.17~0.33	
	150–250	218.7	24	1.57	1.20~1.93		−0.87	−1.09 to −0.66		0.70	0.51~0.87	
	250–350	299.3	14	2.24	1.90~2.68		−0.66	−0.87 to −0.50		1.02	0.87~1.24	
Annual	0	0.0	7	0.96	0.75~1.21	<.01	−1.89	−2.86 to −1.2	.75	0.38	0.29~0.5	<.01
	100–300	246.1	7	1.37	0.77~2.22		−1.42	−2.50 to −0.52		0.60	0.32~0.96	
	300–500	437.9	22	2.67	2.31~3.1		−1.41	−1.92 to −1.02		1.23	1.04~1.45	
	500–700	599.1	11	3.86	3.09~4.57		−1.65	−2.06 to −1.33		1.75	1.38~2.07	

a Indicates the number of observations.

**Figure 3:**
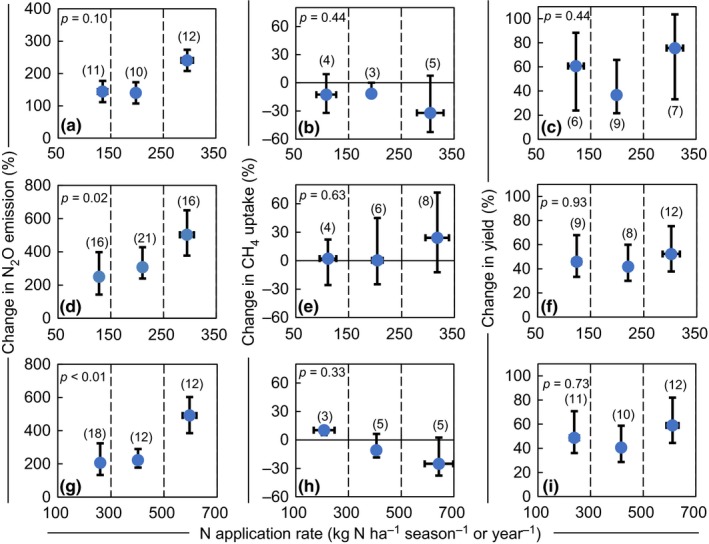
Effect of mineral N application rate on N_2_O emission, CH_4_ uptake, and yield relative to no N fertilizer application for (a–c) wheat season, (d–f) maize season, and (g–i) annual period. Horizontal error bars represent standard errors which reflect distribution of N application rate for each N level. Error bars in vertical directions represent 95% confidence intervals of the percentage changes. Figures in parentheses indicate the number of observations

Relative changes in N_2_O emission remained relatively small at low N application rates, but increased sharply at higher rates (Figure 3a,d,g). This was most evident at annual scale, in which the relative change was as great as 500% under the highest N application rate (500–700 kg N ha^−1^ year ^−1^), nearly twice that under the low N application rate (100–300 kg N ha^−1^ year ^−1^) (*p *<* *.05; Figure 3g). However, N application rates had no significant effect on relative changes of CH_4_ uptake (Figure 3b,e,h), except for low rates (100–300 kg N ha^−1^ year ^−1^) at annual scale, for which the CH_4_ uptake significantly increased, by 10.2% (*p < *.05).

**Figure 4:**
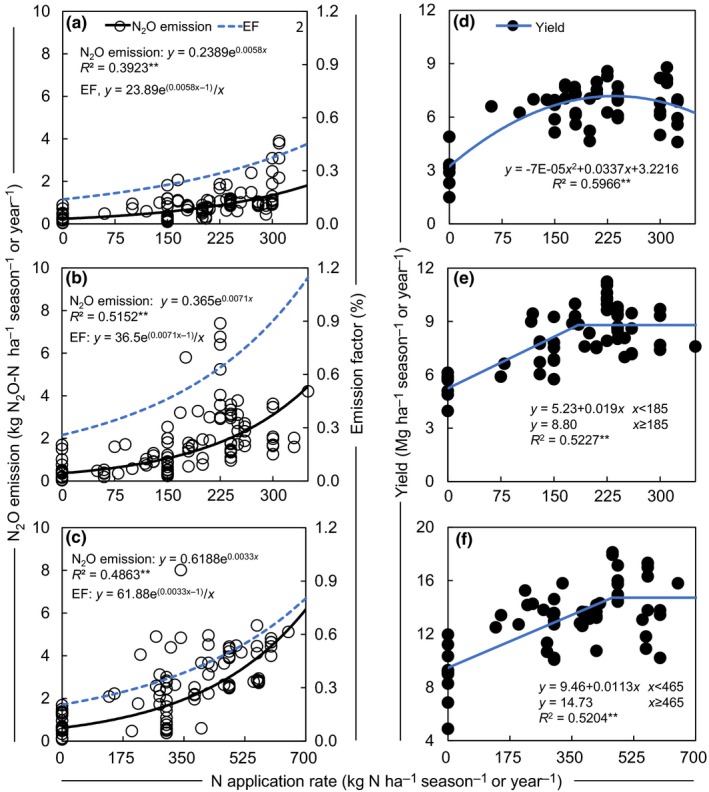
N_2_O emission and emission factor (EF) versus N application rate for (a) wheat season, (b) maize season, and (c) annual period, and yield versus N application rate for (d) wheat season, (e) maize season, and (f) annual period. EF curves were generated from regression models of N_2_O emission with N application rate. ** represents .01 significance level.

Exponential models fit a significant relationship between N_2_O emission and N rate (*p < *.01; Figure 4a–c), especially so for the maize season (*R* ^2^ = 0.52). This indicates that the N_2_O emission increased exponentially in response to increasing N application rate. The EF of N_2_O generated from the exponential model also showed a nonlinear relationship with N application rate (Figure 4a–c). The relationship between crop yield and N application rate could be described by quadratic or linear‐plateau models (*p < *.01; Figure 4d–f). Crop yield maximized at N application rates 241 and 185 kg ha^−1^ season^−1^ (agronomic optimal N rates, AONR) for the wheat and maize seasons, respectively (Figure 4d,e).

### 3.3 Tillage

**Figure 5:**
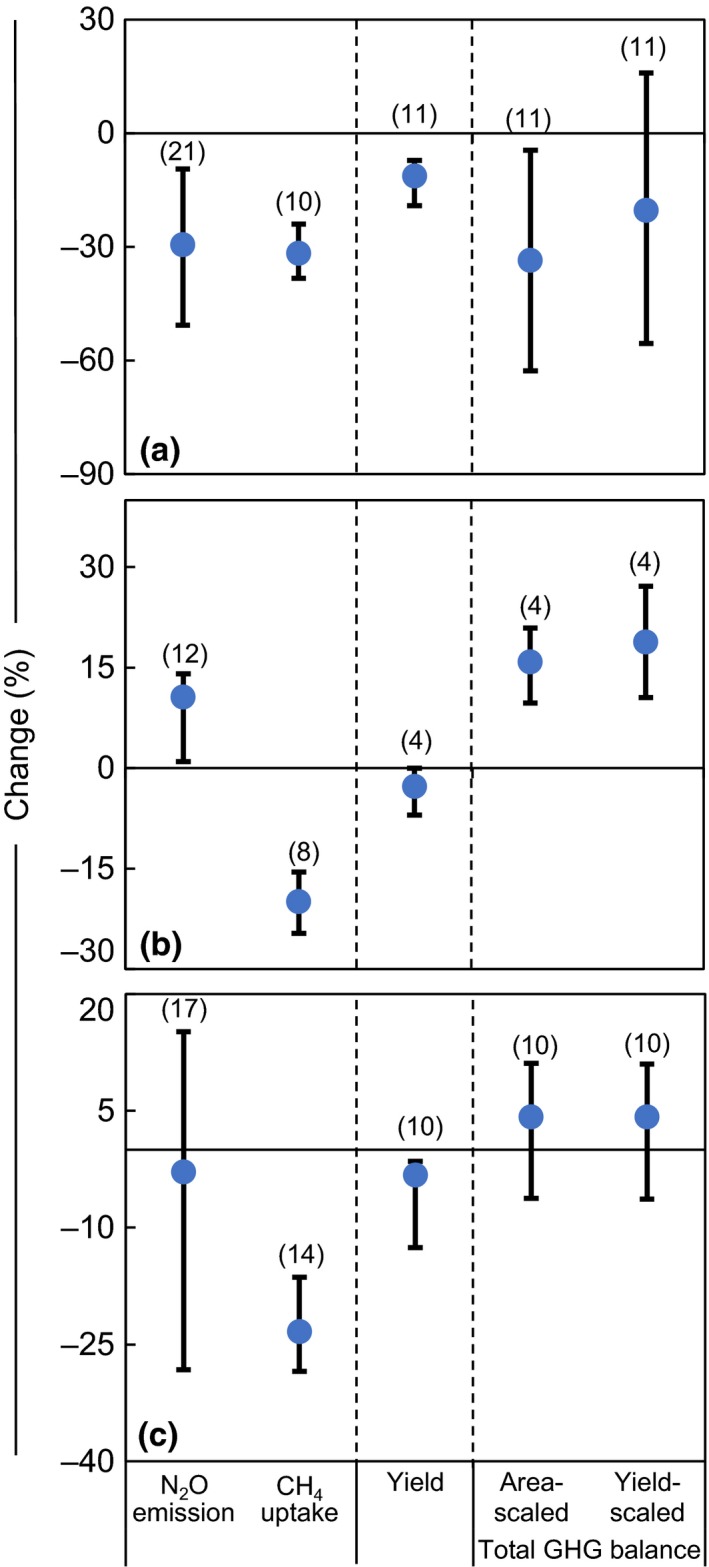
Effect of no‐tillage on N_2_O emission, CH_4_ uptake, yield, and total GHG balance (area‐scaled and yield‐scaled) for (a) wheat season, (b) maize season, and (c) annual period relative to conventional tillage. Data are expressed as mean percentage changes with 95% confidence intervals (represented by error bars). Figures in parentheses indicate number of observations

The effect of tillage on N_2_O emission showed different trends between the wheat and maize seasons. In the wheat season, N_2_O emission significantly declined by nearly 30% under NT (*p < *.05; Figure 5a) as compared with CT. In contrast, N_2_O emission was significantly enhanced (by ~10%) for the maize season (*p < *.05; Figure 5b). At annual scale, there were no significant overall differences in the N_2_O emission (*p* ˃ .05; Figure 5c) between NT and CT management. In contrast, the effect of NT on CH_4_ uptake was consistent between the various growth seasons. Compared with CT, NT significantly (*p *<* *.05) reduced CH_4_ uptake, that is., 31.6%, 19.9%, and 23.3% for the wheat season, maize season, and annual period, respectively (Figure 5a–c).

No‐tillage slightly but significantly decreased crop yield relative to CT (*p < *.05) and was 11.2%, 2.7%, and 3.3% for the wheat season, maize season, and annual period, respectively (Figure 5a–c). The area‐scaled total GHG balances showed similar trends as N_2_O emissions, which decreased significantly by 33% for wheat season (*p < *.05; Figure 5a) and increased significantly by 16% for maize season (*p < *.05; Figure 5b) under NT. However, there was no difference for the annual period (Figure 5c). NT significantly increased yield‐scaled total GHG balance by 18.8% in the maize season (*p *<* *.05; Figure 5b) but had no effect during the wheat season or annually (*p *>* *.05; Figure 5a,c). The similar observations of area‐ and yield‐scaled total GHG balances indicate that the yield decline with NT was not sufficiently large to significantly increase the yield‐scaled total GHG balance. Absolute values for N_2_O emissions under NT were 0.47, 1.46, and 3.51 kg N_2_O‐N ha^−1^ for the wheat season, maize season, and annual period, respectively, and 0.76, 2.38, and 4.01 kg N_2_O‐N ha^−1^ under CT. However, no significant difference was detected between NT and CT (*p > *.05; Table 3). Moreover, there were no significant differences in absolute values of CH_4_ emissions, area‐scaled total GHG balance, or yield between NT and CT (*p > *.05; Table 3).

**Table 3:** N_2_O emission, CH_4_ emission, and area‐scaled total GHG balance for wheat season, maize season, and annual period, as affected by tillage

	Tillage	Obs^a^	N_2_O emission	95% CI	CH_4_ emission	95% CI	Area‐scaled total GHG balance	95% CI	Yield	95% CI
			kg N_2_O‐N ha^−1^		kg CH_4_‐C ha^−1^		Mg CO_2_‐eq ha^−1^		Mg/ha	
Wheat	No‐tillage	6	0.47	0.14~1.01	−0.64	−1.21~0.3	0.19	0.05~0.45	5.13	4.57~5.55
	Tillage	32	0.76	0.57~0.95	−0.45	−0.62~0.24	0.34	0.26~0.43	5.68	4.84~6.61
Maize	No‐tillage	5	1.46	1.15~2.57	−0.86	−1.16~0.61	0.66	0.52~1.17	8.21	6.39~9.11
	Tillage	20	2.38	2.06~2.89	−1.02	−1.29~0.75	1.09	0.94~1.34	7.81	5.97~9.71
Annual	No‐tillage	4	3.51	1.92~4.51	−1.59	−2.53~0.67	1.57	0.71~2.04	13.06	11.04~14.33
	Tillage	20	4.01	3.55~4.51	−1.59	−2.15~1.10	1.87	1.69~2.10	13.63	12.35~14.78

a Indicates the number of observations.

### 3.4 Straw incorporation, application of organic manure, and SRF

**Figure 6:**
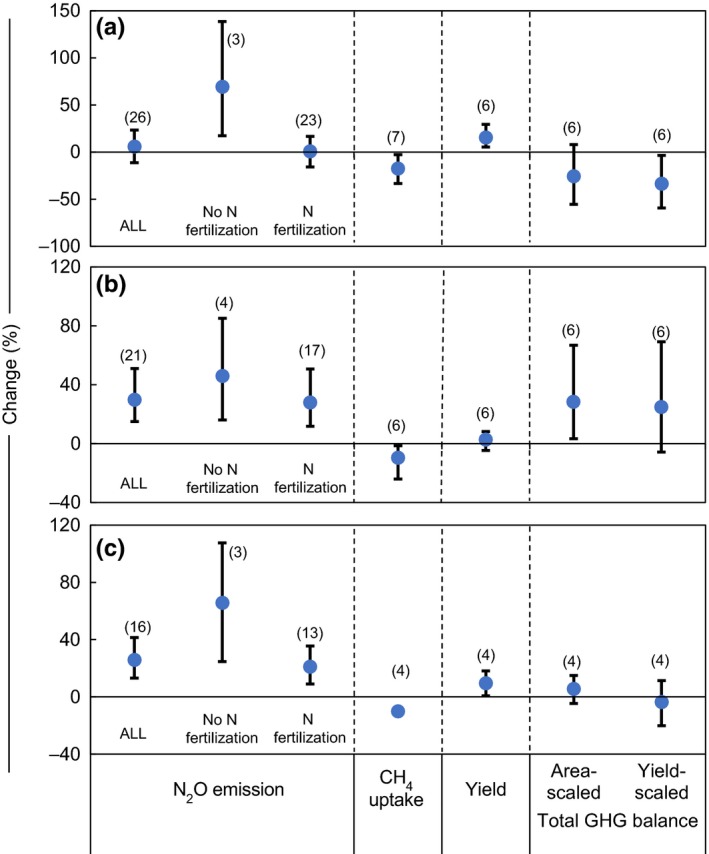
Effect of straw incorporation on N_2_O emission, CH_4_ uptake, yield, and total GHG balance (area‐scaled and yield‐scaled) for (a) wheat season, (b) maize season, and (c) annual period relative to straw removal. Effect sizes for N_2_O emission were separated into no N fertilization and N fertilization. Data are expressed as mean percentage changes with 95% confidence intervals (represented by error bars). Figures in parentheses indicate number of observations

Regardless of N fertilization, N_2_O emission increased with straw incorporation relative to straw removal, especially in maize season (29.9%, *p < *.05; Figure 6b) and the annual period (25.8%, *p < *.05; Figure 6c). The relative increase of N_2_O emission from straw incorporation tended to be greater under no N fertilization as compared with N fertilization. The area‐scaled total GHG balance under straw incorporation significantly increased by 28.4% in maize season (*p < *.05; Figure 6b), but was similar to straw removal in wheat season (*p *>* *.05; Figure 6a). The side‐by‐side comparison showed significant reductions in CH_4_ uptake under straw incorporation compared with straw removal, which were 17.5%, 9.5%, and 10.0% for the wheat season, maize season, and annual period, respectively (*p < *.05; Figure 6a–c). Crop yield under straw incorporation tended to be higher than that under straw removal, especially in wheat season (15.4%) and annual period (25.8%) (Figure 6a,c). This resulted in a decline of yield‐scaled total GHG balance in the wheat season (*p < *.05; Figure 6a). These results indicate that straw incorporation enhanced N_2_O emission and reduced CH_4_ uptake, but achieved a greater crop yield. However, no significant differences in absolute values of N_2_O emission, CH_4_ uptake, area‐scaled total GHG balance, or yield were found between these two straw practices (Table 4).

**Table 4:** N_2_O emission, CH_4_ emission, and area‐scaled total GHG balance for wheat season, maize season, and annual period, as affected by straw management

	Straw management	Obs^a^	N_2_O emission	95% CI	CH_4_ emission	95% CI	Area‐scaled total GHG balance	95% CI	Yield	95% CI
			kg N_2_O‐N ha^−1^		kg CH_4_‐C ha^−1^		Mg CO_2_‐eq ha^−1^		Mg/ha	
Wheat	Incorporation	29	0.74	0.51~0.98	−0.51	−0.69~0.28	0.33	0.22~0.44	5.56	4.57~6.66
	Removal	6	0.59	0.34~0.86	0.29	−0.63~0.10	0.27	0.17~0.39	5.18	4.17~6.43
Maize	Incorporation	15	2.66	2.25~3.29	−1.07	−1.37~0.76	1.21	1.02~1.50	7.91	6.85~8.85
	Removal	4	1.84	1.37~2.97	−0.81	−1.54~0.34	0.84	0.62~1.38	9.11	8.09~10.56
Annual	Incorporation	21	3.99	3.36~4.61	−1.56	−2.08~1.14	1.87	1.59~2.13	13.47	12.08~14.8
	Removal	3	3.54	2.78~4.97	−1.74	−3.27 to −0.69	1.60	1.27~2.13	13.45	13.1~14.92

a Indicates the number of observations.

**Figure 7:**
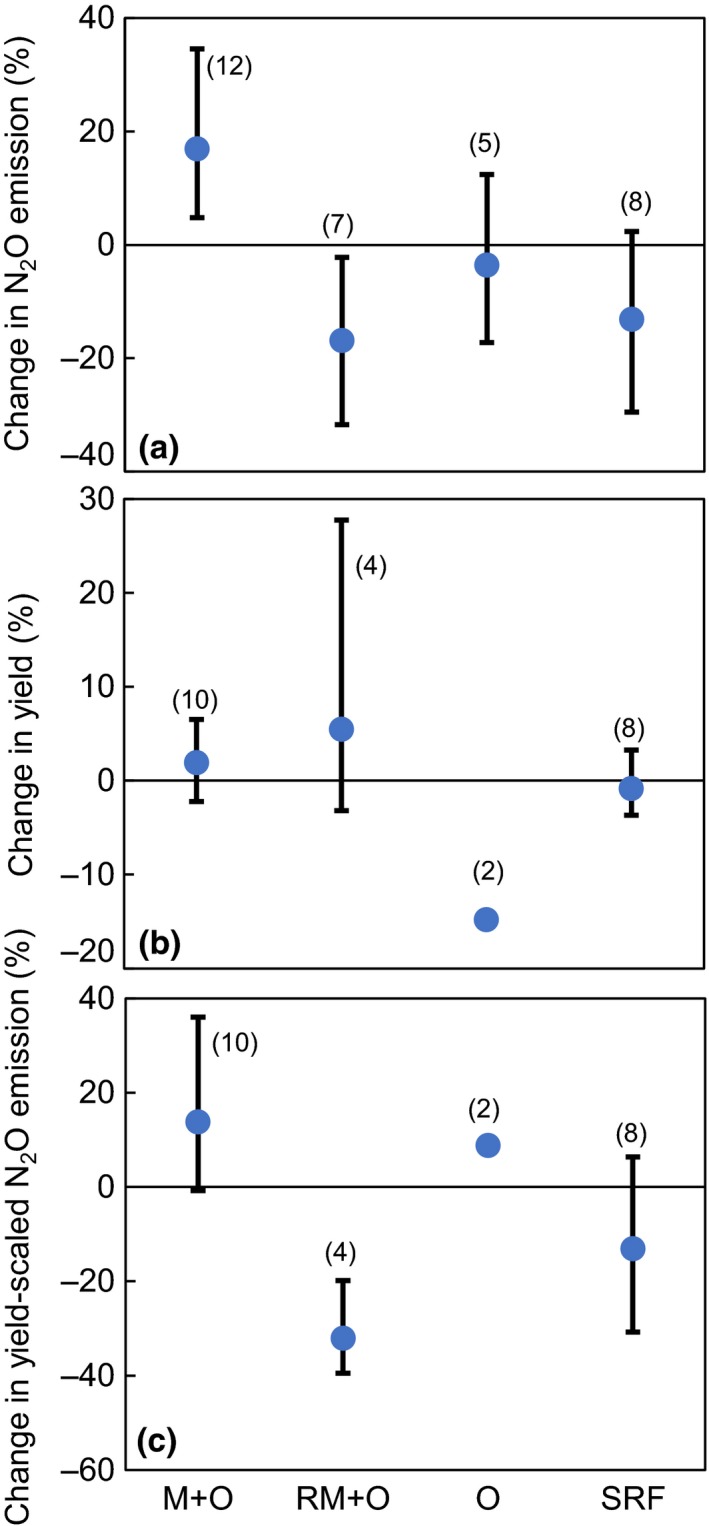
Effect of organic manure and slow‐release fertilizer (SRF) on (a) annual N_2_O emission, (b) yield, and (c) yield‐scaled N_2_O emission relative to mineral fertilizer application alone. M + O and RM + O represent full‐dose and reduced mineral N application rates combined with organic manure, respectively; O represents only organic manure applied. Data are expressed as mean percentage changes with 95% confidence intervals (represented by error bars). Yield‐scaled N_2_O emission represents N_2_O emission per unit crop yield (Mg). Figures in parentheses indicate number of observations

Application of organic manure without mineral fertilizer (O) had no significant effect on N_2_O emission compared to applying mineral fertilizer alone (M) (*p > *.05; Figure 7a), but crop yield declined markedly (14.8%; *p < *.05; Figure 7b). Mixed application of organic manure with full‐dose mineral fertilizer (M+O) significantly increased annual N_2_O emission (by 17.0%) compared with M (*p < *.05; Figure 7a). However, mixed application of organic manure with reduced mineral fertilizer (RM+O, with total N dose equivalent to the M treatment) significantly reduced N_2_O emissions and yield‐scaled N_2_O emissions, by 16.9% and 32.1%, respectively (*p < *.05), while slightly augmenting the crop yields. Compared with M, SRF had no significant effect on either N_2_O emission or yield (*p > *.05; Figure 7a,b).

## 4 DISCUSSION

### 4.1 GHG emission from NCP

Average N_2_O emissions over the NCP (Figure 1) were lower than those of a previous global analysis (Linquist et al., 2012), that is., 0.68 versus 1.44 kg N_2_O‐N ha^−1^ season^−1^ for wheat season and 2.10 versus 3.01 kg N_2_O‐N ha^−1 ^season^−1^ for maize season. A possible reason for this discrepancy is that some studies in the Linquist et al. dataset were of single‐cropping systems (e.g., Grandy, Loecke, Parr, & Robertson, 2006; Parkin & Hatfield, 2010; Parkin & Kaspar, 2006); these have a longer growth period and N_2_O emissions can reach 5.3 and 11.5 kg N_2_O‐N ha^−1^ for the wheat and maize seasons, respectively. Additionally, N_2_O emissions from different climatic zones may also have been distinctly different (Ju et al., 2011). The Linquist et al. dataset included N_2_O emission from a wheat cropping season in South China with a more humid and warmer climate. That emission was as much as 9.29 kg N_2_O‐N ha^−1^ season^−1^, 10 times greater than our findings for the NCP.

In our study, N_2_O emissions were the main contributor (>95%) to the area‐scaled total GHG balance, similar to the findings of Linquist et al. (2012), whereas CH_4_ uptake was negligible. In aerobic soils, CH_4_ is normally oxidized, making these soils sink for atmospheric CH_4_ in dry farmland systems (e.g., Hu et al., 2013; Powlson, Goulding, Willison, Webster, & Hütsch, 1997; Robertson & Grace, 2004). In addition, the radiative forcing potential of N_2_O is ~12 times greater than that of CH_4_ (IPCC, 2007), which has an additional (disproportionate) impact on its estimated contribution to the area‐scaled total GHG balance (Six, Ogle, Conant, Mosier, & Paustian, 2004). These results highlight that GHG mitigation actions in the NCP should mainly target N management and N_2_O.

### 4.2 Natural factors

Heavy rainfall may stimulate N_2_O emission in the NCP (Shi et al., 2013; Yan et al., 2013), but this effect was only observed during maize season in our analysis (Figure 2b). The wheat season in the NCP coincided with very weak precipitation (Wang et al., 2008), so irrigation was more frequently used in that season (~300–500 mm) than in maize season (~100–200 mm). Therefore, the impact of precipitation in wheat season (100–200 mm; Figure 2a) on N_2_O emission could be greatly overwhelmed by sufficient irrigation water.

Effects of soil pH, soil texture, and temperature on N_2_O emission or area‐scaled total GHG balance were also, in most cases, not significant in current study (*p > *.05; Figures 1 and 2d–f). Only one study site (Taian of Shandong Province; Appendix S1) in our database had soil pH <7.4, so pH values in neutral (pH 6.5–7.5) and alkaline soils (pH > 7.5) of the NCP were too similar to produce significant distinctions of GHG emission. Similarly, the narrow range of mean temperature (mostly 7–9°C in wheat season and 24–26°C in maize season; Figure 2d,e) and soil texture (sandy loam to clay loam; Figure 1) across the experimental sites might not have been sufficiently variable to generate significant differences in GHG emission.

### 4.3 Farming practices

#### 4.3.1 N fertilization

The availability of soil N determines N_2_O emissions from soils (Chen et al., 2014; Liu & Zhang, 2011; Van Groenigen, Velthof, Oenema, Van Groenigen, & Van Kessel, 2010). The relative changes of N_2_O emission at low‐to‐moderate N application rates remained relatively constant compared with no N fertilization, but increased sharply at higher N application rates (Figure 3a,d,g). When N is added beyond plant or microorganism demand (Kim, Hernandez‐Ramirez, & Giltrap, 2013; Li et al., 2001), more N remains in the soil, which can then be lost through N_2_O emission (Gerber et al., 2016; Hoben, Gehl, Millar, Grace, & Robertson, 2011; Kim et al., 2013; McSwiney & Robertson, 2005). In our case, the exponential model gave the best fit for the relationship between N_2_O emission and N rate (*p < *.01; Figure 4a–c). There were similar responses of N_2_O emission to N rate observed in crop production fields (Cui et al., 2013; Wang, Chen, Cui, Yue, & Zhang, 2014) and grazed grassland (Cardenas et al., 2010), highlighting the importance of improving N use efficiency toward mitigating N_2_O emissions (Fujinuma, Venterea, & Rosen, 2011; Gagnon, Ziadi, Rochette, Chantigny, & Angers, 2011).

Overuse of N fertilizer may even lead to a decline in crop yield (Ju, Liu, Zhang, & Roelcke, 2004; Liu, Ju, Zhang, Pan, & Christie, 2003; Zhu & Chen, 2002). Our simulation showed that calculated AONR were 241 and 185 kg N ha^−1^ season^−1^ for the wheat and maize season, respectively, with corresponding N_2_O emissions of 0.97 and 1.36 kg N ha^−1^ season^−1^ (Figure 4a,b). Conventional fertilizer N rate of 300 kg N ha^−1^ season^−1^ in the NCP disproportionately increased the N_2_O emission to 1.36 and 3.07 kg N ha^−1^ season^−1^ for the wheat and maize seasons, respectively (Figure 4a,b). This demonstrates that N_2_O emission can be reduced by 0.39 (29%) and 1.71 (56%) kg N_2_O‐N ha^−1^ season^−1^, and a similar crop yield can be maintained under agronomic optimal N rates in the NCP.

The IPCC uses 1% as the default value for EF for upland crops (IPCC, 1997). However, EFs usually are not constant and increase nonlinearly with increasing N rates (Kim et al., 2013; Shcherbak, Millar, & Robertson, 2014). The EFs obtained in our study were 0.37% and 0.90% for the wheat and maize seasons, respectively (Figure 4a,b) at the conventional N rate (300 kg/season), indicating that the 1% default value may overestimate annual N_2_O emissions by ~57% under a conventional N application rate. A previous statistical study also obtained lower EFs than IPCC default value in North China (Shepherd et al., 2015).

#### 4.3.2 Tillage

No‐tillage can result in lower soil temperatures (Linn & Doran, 1984) and higher moisture (Bin et al., 2007; Grandy et al., 2006; Six et al., 2002; Venterea, Maharjan, & Dolan, 2011; Venterea & Stanenas, 2008), which tends to inhibit and enhance N_2_O emissions, respectively. Ding et al. (2007) suggested that N_2_O emission was more sensitive to temperature in wheat season and more affected by soil moisture during maize season. The reduction in N_2_O emission in wheat season and enhancement of N_2_O emission in maize season under NT practice in our study (Figure 5a,b) could have resulted from corresponding changes of temperature and soil moisture as described above. The reduced CH_4_ uptake (*p < *.05; Figure 5a–c) may be explained by the prevention of CH_4_ entering into the soil for CH_4_ oxidation in compacted soil, owing to no‐tillage practice (Omonode, Vyn, Smith, Hegymegi, & Gál, 2007).

Our results also show that annual grain yield under NT was significantly lower than CT (*p *<* *.05; Figure 5c), similar to other meta‐analyses (Kessel et al., 2013; Sainju, Stevens, Caesar‐Tonthat, Liebig, & Wang, 2014; Six et al., 2004; Zhao et al., 2016). The lower grain yield under NT could have been caused by N deficiency (Alvarez & Steinbach, 2009; Ogle, Swan, & Paustian, 2012; Six et al., 2004), cooler soil temperature (Halvorson, Mosier, Reule, & Bausch, 2006), and increased disease pressure (Fernandez et al., 2009). Nevertheless, the risk of yield decline under NT could be minimized by straw return, crop rotation, and other conservation agricultural practices (Zhao et al., 2016).

#### 4.3.3 Straw incorporation

In our study, N_2_O emissions following the incorporation of wheat and maize straw were higher than that under straw removal, particularly in maize season (Figure 6). This was because of increasing anaerobic conditions and enhanced denitrification when straw was returned to soils (Chen, Li, Hu, & Shi, 2013; Mutegi et al., 2010; Shan & Yan, 2013). However, under no N fertilization, the relative increase in N_2_O emission from straw incorporation tended to be greater than under N fertilization (Figure 6). This may be explained by the higher background N_2_O emission in N fertilized soils and the decrease in soil dissolved organic carbon under the combined application of mineral N and crop straw (Liu et al., 2011; Shan & Yan, 2013; Yao et al., 2009). Similarly, straw incorporation can supply substrate and create anaerobic microsites for methanogenesis, which inhibits CH_4_ oxidation (Yao et al., 2013). This is corroborated by our observation that CH_4_ uptake under straw incorporation was significantly reduced by 17.5%, 9.5%, and 10.0% relative to straw removal in the wheat season, maize season, and annual period, respectively (*p < *.05; Figure 6a–c).

Although straw incorporation may induce greater soil‐derived N_2_O emissions, it also promotes soil organic C sequestration (Liu, Lu, Cui, Li, & Fang, 2014; Meng et al., 2016) and avoids substantial, uncontrolled GHG emission from straw burning in the NCP (Lu et al., 2010; Smith et al., 2008). Moreover, we found that annual crop yield under straw incorporation increased significantly by ~9% relative to straw removal (*p < *.05; Figure 6c), similar to a study in Europe (6%; Lehtinen et al., 2014). The impact of straw incorporation on GHG emission should be further comprehensively assessed.

#### 4.3.4 Slow‐release N fertilizer

There have been divergent results of SRF impacts on N_2_O emission, either positive (Akiyama et al., 2013; Li et al., 2015) or negative (Bordoloi & Baruah, 2016; Ji et al., 2013). In the present analysis, SRF reduced annual N_2_O emissions by 13.1%, but this was not statistically significant (*p > *.05; Figure 7a). The effect of SRF on N_2_O emission is modulated by environmental conditions (Hu et al., 2013), the observation period (Hou, Akiyama, Nakajima, Sudo, & Tsuruta, 2000), and crop demand for N (Akiyama, Yan, & Yagi, 2010). Even with no significant reduction in the N_2_O emission, the potential benefits of SRF for reduced NH_3_ volatilization and N leaching should not be neglected (Shaviv & Mikkelsen, 1993).

#### 4.3.5 Mixed application of organic and mineral fertilizer

Compared with M, annual N_2_O emissions significantly increased under M+O (17.0%; *p < *.05; Figure 7a), probably because of the increased supply of C and anaerobic conditions favoring denitrification (Anderson & Levine, 1986; Kamewada, 2007; Velthof, Kuikman, & Oenema, 2003). O appeared to reduce N_2_O emissions but also significantly decreased crop yield (*p < *.05), because of the lack of synchronicity of N supply with crop demand under O treatment (Skinner et al., 2014; Tuomisto, Hodge, Riordan, & Macdonald, 2012). In contrast to M+O, the significant reduction in annual N_2_O emission under RM+O (16.9%; *p < *.05; Figure 7a) was because of lesser N supply from reduced mineral N fertilizer (Yan et al., 2013, 2015). RM+O also slightly increased crop yield (Figure 7c). Hence, application of reduced mineral N with organic manure is a promising alternative farming practice to meet the demands of reducing GHG emissions while maintaining crop yield in the NCP.

### 4.4 Limitations of our analysis

It should be pointed out that literatures reporting N_2_O and CH_4_ emissions and crop production are relatively limited for the NCP, so this may weaken the efficacy of the meta‐analysis. For instance, we cannot reach robust conclusions on tillage, natural factors, and their interaction effects. For the analysis approach, we did a sensitivity analysis that indicated that the weighted and unweighted approaches gave very similar results, for both absolute values and response ratios for GHG emission and crop production as influenced by natural and farming factors. To the best of our knowledge, this study is the first on GHG emissions affected by major farming practices and natural factors in the NCP, which may provide technical support for GHG mitigation in the region.
