# Supplementary material for: Impacts of natural factors and farming practices on greenhouse gas emissions in the North China Plain: A meta‐analysis
Source: Ecol Evol. 2017 Jul 21;7(17):6702–15. doi: 10.1002/ece3.3211 (PMC5587491; doi:10.1002/ece3.3211)
Supplement: Supplementary file 1 [file ECE3-7-6702-s001.docx]

Appendix S1. The collected 76 publications used in this meta-analysis.

| Reference | Site | Coordinate | Soil pH | Soil texture | Cropping season | N_2_O | CH_4_ | Yield | Farming practice |
| --- | --- | --- | --- | --- | --- | --- | --- | --- | --- |
| (Cai *et al.*, 2011) | Fengqiu,  Henan Province | 35°00′N, 114°24′ E | 8.6 | Sandy loam | M | Y | N | N | Tillage |
| (Cai *et al.*, 2012) | Fengqiu,  Henan Province | 35°00´N, 114°24´E | 8.5 | Sandy loam | M | Y | N | Y | N fertilization/OF |
| (Cai *et al.*, 2013) | Fengqiu,  Henan Province | 35°00´N, 114°24´E | 8.5 | Sandy loam | W/M/W-M | Y | N | N | N fertilization/OF |
| (Cui *et al.*, 2012) | Zibo,  Shandong Province | 36°58´N, 117°59´E | 8.3 | Silty loam | W/M/W-M | Y | N | N | N fertilization |
| (Ding *et al.*, 2007) | Fengqiu,  Henan Province | 35°00′N, 114°24′E | 8.7 | Sandy loam | W/M/W-M | Y | N | N | N fertilization |
| (Ding *et al.*, 2011) | Fengqiu,  Henan Province | 35°00′N, 114°24´E | 8.6 | Sandy loam | M | Y | N | Y | N fertilization |
| (Ding *et al.*, 2013) | Fengqiu,  Henan Province | 35°00´N, 114°24´E | 8.0 | Sandy loam | W/M/W-M | Y | N | N | N fertilization/OF |
| (Ding *et al.*, 2015) | Fengqiu,  Henan Province | 35°00´N, 114°24´E | 8.6 | Sandy loam | W | Y | N | Y | N fertilization |
| (Gao, 2004) | Beijing | 39°34′N, 116°18′E | 8.0 | Sandy loam | W/M/W-M | Y | Y | Y | N fertilization/straw management |
| (Gao *et al.*, 2005) | Beijing | 39°30′N, 116°18′E | 8.0 | Silty loam | M | Y | N | N | N fertilization |
| (Gao *et al.*, 2014) | Handan,  Hebei Province | 36°52´N, 115°10´E | 7.7 | Silty loam | W/M/W-M | Y | Y | Y | N fertilization/straw management |
| (Hu, 2011) | Handan,  Hebei Province | 36°52′N, 115°10′E | 8.2 | Silty loam | W/M/W-M | Y | Y | N | N fertilization/SRF |
| (Hu *et al.*, 2011) | Handan,  Hebei Province | 36°52′N, 115°10′ E | 8.2 | Silty loam | M | Y | Y | Y | N fertilization/straw management/SRF |
| (Hu *et al.*, 2013) | Handan,  Hebei Province | 36°52´N, 115°10´E | 8.2 | Silty loam | W/M/W-M | Y | Y | Y | N fertilization/straw management/SRF/OF |
| (Huang *et al.*, 2011) | Shijiazhuang,  Hebei Province | 37°50′ N, 114°40′E | 8.5 | Sandy loam | W | Y | N | N | Tillage |
| (Huang *et al.*, 2013) | Beijing | 39°48´N, 116°28´E | 8.1 | Clay loam | W/M/W-M | Y | N | Y | N fertilization/tillage |
| (Huang, 2014) | Beijing | 39°48´N, 116°28´E | 8.1 | Clay loam | W/M/W-M | Y | N | Y | N fertilization/straw management/OF |
| (Ju *et al.*, 2011) | Beijing | 40°04′N, 116°16′E | 7.9 | Sandy loam | M | Y | N | N | N fertilization |
| (Li, 2007) | Shijiazhuang,  Hebei Province | 37°50′N, 114°40′E | 8.5 | Sandy loam | W/M/W-M | Y | Y | N | Straw management/tillage |
| (Li, 2013) | Taian,  Shandong Province | 36°10´N, 117°09´E | 5.9 | Loam | M | Y | Y | Y | N fertilization |
| (Li, 2014a) | Taian,  Shandong Province | 36°18′N, 117°12′E |  | Loam | M | Y | N | Y | N fertilization/tillage/SRF |
| (Li, 2014b) | Beijing | 39°48´N, 116°28´E | 8.0 | Clay loam | W/M/W-M | Y | Y | N | N fertilization |
| (Li *et al.*, 2010) | Handan,  Hebei Province | 36°52´N, 115°10´E | 7.8 | Silty loam | W-M | Y | N | N | N fertilization |
| (Li *et al.*, 2015a) | Dezhou,  Shandong Province |  |  | Loam | M | Y | Y | N | Straw management/OF |
| (Li *et al.*, 2015b) | Dezhou,  Shandong Province | 37°20′N, 116°38′E |  | Loam | M | Y | N | Y | N fertilization/OF |
| (Li *et al.*, 2016) | Dezhou,  Shandong Province |  |  | Loam | W | Y | Y | N | Straw management/OF |
| (Liang, 2009) | Yongji,  Shanxi Province | 34°56′N, 110°43′E | 8.5 | Clay loam | M | Y | Y | N | Straw management |
| (Liu, 2013) | Zibo,  Shandong Province | 36°59´N, 117°60´E | 8.1 | Sandy loam | W/M/W-M | Y | Y | Y | N fertilization/SRF/OF |
| (Liu *et al.*, 2011) | Yongji,  Shanxi Province | 34°55´N, 110°42´E | 8.7 | Clay loam | W/M/W-M | Y | N | Y | N fertilization/straw management |
| (Liu *et al.*, 2012) | Yongji,  Shanxi Province | 34°55´N, 110°42´E | 8.7 | Clay loam | W/M/W-M | Y | Y | Y | N fertilization |
| (Liu *et al.*, 2014) | Yongji,  Shanxi Province | 34°55´N, 110°42´E | 8.7 | Clay loam | W/M/W-M | Y | N | Y | N fertilization |
| (Long, 2014) | Cangzhou,  Hebei Province | 37°41´N, 116°37´E | 8.2 | Silty loam | W/M/W-M | Y | Y | Y | N fertilization/OF |
| (Ma *et al.*, 2012) | Baoding,  Hebei Province | 38°08′N, 115°4′E | 8.6 | Silty clay loam | M | Y | N | N | N fertilization |
| (Meng *et al.*, 2005a) | Fengqiu,  Henan Province | 35°00′N, 114°24′ E | 8.7 | Silty loam | W-M | Y | N | N | N fertilization/OF |
| (Meng *et al.*, 2005b) | Fengqiu,  Henan Province | 35°00´N, 114°24′E | 8.7 | Sandy loam | W/M/W-M | Y | N | N | N fertilization/OF |
| (Meng *et al.*, 2008) | Fengqiu,  Henan Province | 35°00′N, 114°24′ E | 8.7 | Sandy loam | W/M/W-M | Y | N | N | N fertilization/OF |
| (Pei, 2012) | Baoding,  Hebei Province | 38°71N, 115◦15′E | 8.1 | Sandy loam | W/M/W-M | Y | Y | Y | N fertilization/straw management |
| (Pei *et al.*, 2012a) | Baoding,  Hebei Province | 38°71′N, 115°15′E | 8.1 | Sandy loam | M | Y | N | N | N fertilization/straw management |
| (Pei *et al.*, 2012b) | Baoding,  Hebei Province | 38°71′N, 115◦15′E | 8.1 | Sandy loam | W/M/W-M | Y | N | N | N fertilization/straw management |
| (Qiu, 2012) | Zibo,  Shandong Province | 36°52′N, 118°10′E | 8.8 | Sandy loam | W-M | Y | Y | Y | N fertilization/SRF/OF |
| (Shi, 2012) | Baoding,  Hebei Province | 38°48´N, 115°24´E | 8.4 | Silty clay loam | M | Y | Y | Y | N fertilization/SRF |
| (Shi, 2013) | Zibo,  Shandong Province | 36°58´N, 117°59´E | 7.7 | Sandy loam | M/W-M | Y | Y | Y | N fertilization/tillage/SRF |
| (Shi *et al.*, 2013) | Zibo,  Shandong Province | 36°59´N, 117°60´E | 7.4 | Sandy loam | W/M/W-M | Y | Y | Y | N fertilization/straw management/tillage/SRF/OF |
| (Shi *et al.*, 2014) | Zibo,  Shandong Province | 36°58´N, 117°59´E | 7.7 | Sandy loam | M | Y | Y | Y | N fertilization/SRF/OF |
| (Song & Zeng, 1997) | Shijiazhuang,  Hebei Province | 37°53′N, 114°41′E | 8.5 | Sandy loam | W-M | Y | N | N | N fertilization/OF |
| (Song *et al.*, 2013) | Shijiazhuang,  Hebei Province | 37°53′N, 114°41′E | 8.5 | Sandy loam | W | Y | Y | N | N fertilization/tillage |
| (Sun *et al.*, 2008) | Shijiazhuang,  Hebei Province | 36°57′N, 116°38′E | 7.9 | Sandy loam | W/M/W-M | Y | N | N | N fertilization |
| (Tian, 2010) | Taian,  Shandong Province | 36°09′N, 117°09′E | 6.8 | Loam | W/M/W-M | Y | Y | Y | Straw management/tillage |
| (Tian *et al.*, 2012) | Taian,  Shandong Province | 36°09′N, 117°09′E | 6.2 | Loam | W | Y | Y | Y | Straw management/tillage |
| (Tian *et al.*, 2013) | Taian,  Shandong Province | 36°09′N, 117°09′E | 6.8 | Loam | W-M | Y | Y | Y | Tillage |
| (Wan *et al.*, 2008) | Shijiazhuang,  Hebei Province | 37°50′N, 114°40′E | 7.8 | Sandy loam | W | Y | N | N | N fertilization/straw management |
| (Wan *et al.*, 2009) | Shijiazhuang,  Hebei Province | 37°53′N, 114°41′E | 8.5 | Sandy loam | W | Y | Y | Y | Straw management/OF |
| (Wang & Hu, 2011) | Shijiazhuang,  Hebei Province | 37°53′N, 114°41′E | 8.5 | Sandy loam | M | Y | Y | N | N fertilization |
| (Wang *et al.*, 2008) | Handan,  Hebei Province | 36°52′N, 115°01′E | 7.8 | Silty loam | W-M | Y | N | N | N fertilization |
| (Wang *et al.*, 2009) | Shijiazhuang,  Hebei Province | 37°50′N, 114°40′E | 8.5 | Sandy loam | W/M/W-M | Y | Y | Y | N fertilization |
| (Wang *et al.*, 2015) | Baoding,  Hebei Province |  | 7.7 |  | W/M/W-M | Y | N | Y | N fertilization |
| (Wei *et al.*, 2012) | Shijiazhuang,  Hebei Province | 37°50′ N, 114°40′E | 8.5 | Sandy loam | W-M | Y | Y | N | Straw management/tillage |
| (Xia *et al.*, 2014) | Xinxiang,  Henan Province | 35°09´N, 113°51´E | 8.1 | Sandy loam | M | Y | Y | Y | Straw management |
| (Xiao *et al.*, 2011) | Shijiazhuang,  Hebei Province | 37°50′ N, 114°40′E | 8.5 | Sandy loam | W-M | Y | Y | Y | N fertilization |
| (Xu *et al.*, 2000) | Fengqiu,  Henan Province | 35°00′N, 114°24′ E | 9.0 | Sandy loam | W | Y | N | N | N fertilization |
| (Xu *et al.*, 2015) | Dezhou,  Shandong Province | 36°57′N, 116°38′E | 8.4 | Silty loam | W | Y | N | Y | Straw management/tillage |
| (Xu *et al.*, 2016) | Taian,  Shandong Province | 36°09′N, 117°15′E | 7.8 | Loam | W | Y | Y | Y | Straw management/SRF |
| (Yan *et al.*, 2013) | Zibo,  Shandong Province | 36◦58′N, 117◦59′E | 8.3 | Sandy loam | W-M | Y | N | N | N fertilization |
| (Yan *et al.*, 2015) | Zibo,  Shandong Province | 36◦58′ N, 117°59′E | 8.3 | Silty loam | W-M | Y | N | Y | N fertilization/SRF/OF |
| (Zeng *et al.*, 1995) | Shijiazhuang,  Hebei Province | 37°53′N, 114°41′E | 8.5 | Sandy loam | W-M | Y | N | N | N fertilization/OF |
| (Zhang, 2006) | Baoding,  Hebei Province | 39.95°N,  116.3°E | 8.4 |  | W/M/W-M | Y | N | N | N fertilization/OF |
| (Zhang, 2009) | Shijiazhuang,  Hebei Province | 37°50′N, 114°40′E | 8.5 | Sandy loam | W/M/W-M | Y | Y | Y | Straw management/tillage |
| (Zhang *et al.*, 2006) | Baoding,  Hebei Province | 39°57′N, 116°18′E | 8.4 |  | M | Y | N | N | N fertilization/OF |
| (Zhang *et al.*, 2011) | Baoding,  Hebei Province | 38◦71′N, 115◦15′E | 8.1 | Sandy loam | M | N | N | N | N fertilization/straw management |
| (Zhang *et al.*, 2012) | Baoding,  Hebei Province | 38◦71′N, 115◦15′E | 8.1 | Sandy loam | M | Y | N | N | N fertilization/straw management/OF |
| (Zhang *et al.*, 2013) | Xinxiang,  Henan Province | 35°18′N, 113°54′E | 8.3 | Loam | W | Y | N | Y | Straw management/tillage/OF |
| (Zhang *et al.*, 2016) | Taian,  Shandong Province | 36°11′N, 117°08′E | 7.0 | Clay loam | W/M/W-M | Y | N | Y | N fertilization/SRF |
| (Zhao, 2008) | Taian,  Shandong Province | 36°09′N, 117°09′E | 6.8 | Loam | W/M/W-M | Y | Y | Y | Straw management/tillage |
| (Zhao *et al.*, 2008a) | Taian,  Shandong Province | 36°10′N, 117°09′E | 6.8 | Loam | W | Y | N | N | Straw management/tillage |
| (Zhao *et al.*, 2008b) | Taian,  Shandong Province | 36°10′N, 117°09′E | 6.8 | Loam | W | N | Y | N | Straw management/tillage |
| (Zhou *et al.*, 2016) | Baoding,  Hebei Province | 38°71′N, 115°15′E | 8.7 | Sandy loam | M | Y | N | Y | N fertilization |

W, M and W-M represent winter wheat, summer maize growing season and whole wheat-maize rotation, respectively; OF, organic fertilizer; SRF, slow-release fertilizer; Y, yes; N, no.

**References**

Cai Y, Ding W, Luo J (2012) Spatial variation of nitrous oxide emission between interrow soil and interrow plus row soil in a long-term maize cultivated sandy loam soil. Geoderma*,* **181-182**, 2-10.

Cai Y, Ding W, Luo J (2013) Nitrous oxide emissions from Chinese maize-wheat rotation systems: A 3-year field measurement. Atmospheric Environment*,* **65**, 112-122.

Cai YJ, Ding WX, Zhu AN, Zhang JB (2011) Effects of non-tillage on N_2_O and CO_2_ emissions from sandy loam soil in the North China Plain. Journal of Ecology and Rural Environment*,* **27**, 1-6.

Cui F, Yan G, Zhou Z, Zheng X, Deng J (2012) Annual emissions of nitrous oxide and nitric oxide from a wheat-maize cropping system on a silt loam calcareous soil in the North China Plain. Soil Biology and Biochemistry*,* **48**, 10-19.

Ding W, Cai Y, Cai Z, Yagi K, Zheng X (2007) Nitrous oxide emissions from an intensively cultivated maize-wheat rotation soil in the North China Plain. Science of Total Environment*,* **373**, 501-511.

Ding W, Luo J, Li J, Yu H, Fan J, Liu D (2013) Effect of long-term compost and inorganic fertilizer application on background N_2_O and fertilizer-induced N_2_O emissions from an intensively cultivated soil. Science of Total Environment*,* **465**, 115-124.

Ding WX, Chen ZM, Yu HY *et al.* (2015) Nitrous oxide emission and nitrogen use efficiency in response to nitrophosphate, N-(n-butyl) thiophosphoric triamide and dicyandiamide of a wheat cultivated soil under sub-humid monsoon conditions. Biogeosciences*,* **12**, 803-815.

Ding WX, Yu HY, Cai ZC (2011) Impact of urease and nitrification inhibitors on nitrous oxide emissions from fluvo-aquic soil in the North China Plain. Biology and Fertility of Soils*,* **47**, 91-99.

Gao B, Ju X, Su F *et al.* (2014) Nitrous oxide and methane emissions from optimized and alternative cereal cropping systems on the North China Plain: a two-year field study. Science of Total Environment*,* **472**, 112-124.

Gao ZL (2004) N_2_O flux and CH_4_ uptake in soil of winter wheat and summer maize rotation system. PhD thesis, China Agricultrual University. (in Chinese with English abstract).

Gao ZL, Chen XP, Zhang FS, Kogge M, Romheld V (2005) Continous-automatic method for measuring N_2_O emission from agricultural soil. Plant Nutrition and Fertilizer Science*,* **11**, 64-70 (in Chinese with English abstract).

Hu XK, Su F, Ju XT *et al.* (2013) Greenhouse gas emissions from a wheat-maize double cropping system with different nitrogen fertilization regimes. Environmental Pollution*,* **176**, 198-207.

Hu XK (2011) Greenhouse gases fluxes of winter wheat-summer maize rotation and mitigation strategies on the North China Plain. PhD thesis, China Agricultrual University. (in Chinese with English abstract).

Hu XK, Huang BX, Su F, Ju XT. (2011) Effects of nitrogen management on methane and nitrous oxide emissions from summer maize soil in North China Plain. SCIENTIA SINICA Chimica*,* **41**, 117-128 (in Chinese with English abstract).

Huang GH, Zhang MY, Chen F, Zhang HL (2011) Influences of tillage methods on N_2_O emission from winter wheat field in North China Plain. Transactions of the CSAE*,* **27**, 167-173 (in Chinese with English abstract).

Huang T (2014) The effects of long-term C and N inputs on soil organic C and N pools and environments. PhD thesis, China Agricultrual University. (in Chinese with English abstract).

Huang T, Gao B, Christie P, Ju X (2013) Net global warming potential and greenhouse gas intensity in a double-cropping cereal rotation as affected by nitrogen and straw management. Biogeosciences*,* **10**, 7897-7911.

Ju X, Lu X, Gao Z *et al.* (2011) Processes and factors controlling N_2_O production in an intensively managed low carbon calcareous soil under sub-humid monsoon conditions. Environmental Pollution*,* **159**, 1007-1016.

Li L (2007) Influence of conservation tillage on soil organic carbon pool and greenhouse gases emission. PhD thesis, China Agricultrual University. (in Chinese with English abstract).

Li H, Qiu JJ, Wang LG, Tang HJ, Li CS, Van Ranst E (2010) Modelling impacts of alternative farming management practices on greenhouse gas emissions from a winter wheat-maize rotation system in China. Agriculture, Ecosystems and Environment*,* **135**, 24-33.

Li N (2014a) Effects of tillage practice and poly-coated urea on N_2_O from summer maize field. Master thesis, Shandong Agricultural University. (in Chinese with English abstract).

Li X (2013) Effects of biochar and biochar-based fertilizer anmendment on greenhouse gas emission, maize growth and soil properties. Master thesis, Nanjing Agricultural University. (in Chinese with English abstract).

Li XH, Zhu ZL, Dong HY, Yang LP, Guo HH (2015a) Effects of different return modes of wheat straws on greenhouse gas emissions and carbon sequestration of maize fields. Journal of Agro-Environment Science*,* **34**, 2228-2235.

Li XH, Zhu ZL, Dong HY, Yang LP, Guo HH (2016) Characteristics of greenhouse gas emissions from the wheat fields with different returning methods of maize straws. Journal of Agricultural Resources and Environment*,* **33**, 176-181 (in Chinese with English abstract).

Li XM (2014b) Study on greenhouse gas emissions and its influencing factors from the maize-wheat rotation field. Master thesis, Beijing Forestry University. (in Chinese with English abstract).

Li YQ, Tang JW, Che SG, Wen Y, C., Sun WY, Zhao BQ (2015b) Effect of organic and inorganic fertilizer on the emission of CO_2_ and N_2_O from the summer maize field in the North China Plain. Scientia Agricultura Sinica, 4381-4389 (in Chinese with English abstract).

Liang WG (2009) Study of impacts of amendant plant residues on the emissions of greenhouse gases and NO from typical saline soil. Master thesis, Southwest University. (in Chinese with English abstract).

Liu C, Wang K, Meng S *et al.* (2011) Effects of irrigation, fertilization and crop straw management on nitrous oxide and nitric oxide emissions from a wheat-maize rotation field in northern China. Agriculture, Ecosystems & Environment*,* **140**, 226-233.

Liu C, Wang K, Zheng X (2012) Responses of N_2_O and CH_4_ fluxes to fertilizer nitrogen addition rates in an irrigated wheat-maize cropping system in northern China. Biogeosciences*,* **9**, 839-850.

Liu C, Yao Z, Wang K, Zheng X (2014) Three-year measurements of nitrous oxide emissions from cotton and wheat-maize rotational cropping systems. Atmospheric Environment*,* **96**, 201-208.

Liu DX (2013) Optimal fertilization reduced greenhouse gas emissions of wheat-maize cropping system. Master thesis, Shandong Agricultural University. (in Chinese with English abstract).

Long P (2014) Effects of organic wastes incorporation on soil organic carbon and net carbon balance in wheat-maize farming system. PhD thesis, China Agricultrual University. (in Chinese with English abstract).

Ma YL, Ji YZ, Li X, Zhang L, Ju X, T., Zhang LJ (2012) Effects of N fertilization rates on the NH_3_ volatilization and N_2_O emissions from the wheat-maize rotation system in North China Plain. Ecology and Environmental Sciences*,* **21**, 225-230 (in Chinese with English abstract).

Meng L, Cai ZC, Ding WX (2005a) Carbon contents in soils and crops as affected by long-term fertilization. ACTA PEDOLOGICA SINICA*,* **42**, 769-776 (in Chinese with English abstract).

Meng L, Cai ZC, Ding WX (2008) Effects of long-term fertilization on N distribution and N_2_O emission in fluvoaquci soil in North China. ACTA ECOLOGICA SINICA*,* **28**, 6197-6203 (in Chinese with English abstract).

Meng L, Ding W, Cai Z (2005b) Long-term application of organic manure and nitrogen fertilizer on N_2_O emissions, soil quality and crop production in a sandy loam soil. Soil Biology and Biochemistry*,* **37**, 2037-2045.

Pei SW (2012) The effect of fertilization on greenhouse gas and nitrogen gas emissions from the field in North China Plain. Master thesis, Beijing Forestry University. (in Chinese with English abstract).

Pei SW, Zhang YY, Liu JF, J. MY, Lun XX (2012a) Greenhouse gas emission under the treatments of fertilization and wheat straw returning during the maize growing seasons. Environmental Chemistry*,* **31**, 407-414 (in Chinese with English abstract).

Pei SW, Zhang YY, Liu JF, Lun XX, Mu YJ (2012b) N_2_O exchange fluxes from wheat-maize crop rotation system in the North China Plain. Environmental Science*,* **33**, 3641-3646 (in Chinese with English abstract).

Qiu JJ (2012) *Research on the assessment and regulation of Carbon and Nitrogen balance in circum-Bohai-sea agricultural region*, Science Press, Beijing, China.

Shi SJ (2012) Typical farmland N_2_O and CH_4_ net exchange characteristics of different nitrogen management. Master thesis, Hebei Agricultural University. (in Chinese with English abstract).

Shi YF, Wu WL, Meng FQ, Zheng L, Wang DP, Ye H, Ding GW (2014) Nitrous oxide and methane fluxes during the maize season under optimized management in intensive farming systems of the North China Plain. Pedosphere*,* **24**, 487-497.

Shi Y, Wu W, Meng F, Zhang Z, Zheng L, Wang D (2013) Integrated management practices significantly affect N_2_O emissions and wheat-maize production at field scale in the North China Plain. Nutrient Cycling In Agroecosystems*,* **95**, 203-218.

Shi YF (2013) Greenhouse gases emissions of crop production systems in the high yield regions of the North China Plain. PhD thesis, China Agricultural University. (in Chinese with English abstract).

Song LN, Zhang YM, Hu CS, Zhang XY, Dong WX, Wang YY, Qin SP (2013) Comprehensive analysis of emissions and global warming effects of greenhouse gases in winter-wheat fields in the high-yield agro-region of North China Plain. Chinese Journal of Eco-Agriculture*,* **21**, 297-307 (in Chinese with English abstract).

Song WZ, Zeng JH (1997) Emissions of nitrous oxide from dryland in Northern China. ANVANCES IN ENVIRONMENTAL SCIENCE, 49-55 (in Chinese with English abstract).

Sun YL, Lu PL, Li J, Yu Q, Sun SB, Wang JS, Ouyang Z (2008) Characteristics of soil N_2_O flux in a winter wheat-summer maize rotation system in North China Plain and analysis of influencing factors. Chinese Journal of Agrometeorology*,* **29**, 1-5 (in Chinese with English abstract).

Tian S, Ning T, Zhao H *et al.* (2012) Response of CH_4_ and N_2_O emissions and wheat yields to tillage method changes in the north China plain. Plos One*,* **7**, e51206.

Tian S, Wang Y, Ning T *et al.* (2013) Greenhouse gas flux and crop productivity after 10 years of reduced and no tillage in a wheat-maize cropping system. Plos One*,* **8**, e73450.

Tian SZ (2010) Effects of tillage measures and conversion on the emission of soil CH_4_, N_2_O and carbon sequestration capability in wheat-maize farmland. Master thesis, Shandong Agricultural University. (in Chinese with English abstract).

Wan YF, Li YE, Gao QZ, Liu YT, Qin XB (2008) Characteristics of N_2_O flux in winter wheat field under different field managements. Chinese Journal of Agrometeorology*,* **29**, 130-133 (in Chinese with English abstract).

Wan YF, Li YE, Gao QZ, Qin XB, Lin ED (2009) Field managements affect yield, soil carbon, and greenhouse gases emission of winter wheat in North China Plain. Journal of Agro-Environment Science*,* **28**, 2495-2500 (in Chinese with English abstract).

Wang LG, Li H, Qiu JJ (2008) Characterization of emissions of nitrous oxide from soils of typical crop fields in Huang-Huai-Hai Plain. Scientia Agricultura Sinica*,* **41**, 1248-1254 (in Chinese with English abstract).

Wang YQ, Li YC, Peng ZP, Wang CN, Liu YN (2015) Effects of dicyandiamide combined with nitrogen fertilizer on N_2_O emission and economic benefit in winter wheat and summer maize rotation system. Chinese Journal of Applied Ecology*,* **26**, 1999-2006 (in Chinese with English abstract).

Wang YY, Hu CS (2011) Soil greenhouse gas emission in winter wheat/summer maize rotation ecosystem as affected by nitrogen fertilization in the Piedmont Plain of Mount Taihang, China. Chinese Journal of Eco-Agriculture*,* **19**, 1122-1128 (in Chinese with English abstract).

Wang YY, Hu CS, Cheng YS, Zhang YM, Ming H, Yang PP (2009) Carbon sequestrations and gas regulations in summer-maize and winter-wheat rotation ecosystem affected by nitrogen fertilization in the piedmont plain of Taihang mountains, China. Journal of Agro-Environment Science*,* **28**, 1508-1515 (in Chinese with English abstract).

Wei YH, Zhang EP, Chen F, Zhang Y, Zhang HL (2012) Effects of tillage systems on greenhouse gas emission of wheat-maize double cropping system in North China Plain. Advanced Materials Research*,* **524-527**, 2526-2532.

Xia WB, Zhang XH, Liu ML, Pan GX, Zheng JF, Li L, Q., Zheng JW (2014) Effects of wheat straw return ways on integrated global warming effect from dryland soil in North China Plain. Soils, 1010-1016 (in Chinese with English abstract).

Xiao Y, Xie GD, An K, Liu CL, Chen CC (2011) Ecosystem services of wheat-maize cropland systems in the North China Plain. Chinese Journal of Eco-Agriculture*,* **19**, 429-435 (in Chinese with English abstract).

Xu H, Xing GX, Cai ZC, Hetian ZX (2000) Effect of soil texture on N_2_O emissions from winter wheat and cotton fields. Agro-environmental Protection*,* **19**, 1-3 (in Chinese with English abstract).

Xu Y, Liu ZH, Zhu GL, Li X, Tan DS, Shi J, Jiang LH (2016) Effects of greenhouse gas emission under different agricultural management practices in wheat field in the North China Plain. Soil and Fertilizer Sciences in China, 7-13 (in Chinese with English abstract).

Xu YY, Wu LF, Li BB, Qiu QY, Qin Y (2015) Soil N_2_O emission and its agronomic efficiency under different tillage. Chinese Journal of Eco-Agriculture*,* **23**, 1349-1358 (in Chinese with English abstract).

Yan G, Yao Z, Zheng X, Liu C (2015) Characteristics of annual nitrous and nitric oxide emissions from major cereal crops in the North China Plain under alternative fertilizer management. Agriculture, Ecosystems & Environment*,* **207**, 67-78.

Yan G, Zheng X, Cui F, Yao Z, Zhou Z, Deng J, Xu Y (2013) Two-year simultaneous records of N_2_O and NO fluxes from a farmed cropland in the northern China plain with a reduced nitrogen addition rate by one-third. Agriculture, Ecosystems & Environment*,* **178**, 39-50.

Zeng JH, Wang ZP, Zhang YM, Song WZ, Wang SB, Su WH (1995) Flux of N_2_O emission from fields in a wheat and maize rotation system. Chinese Journal of Environmental Science, 32-210 (in Chinese with English abstract).

Zhang H, Guo LP, Xie LY, Lin M, Ye DD, Yan HL (2013) The effect of management practices on the emission of CO_2_ and N_2_O from the winter wheat field in North China Plain. Chinese Journal of Soil Science, 653-659 (in Chinese with English abstract).

Zhang J, Xia GL, Li H *et al.* (2016) Effect of single basal fertilization on N_2_O emissions in wheat and maize rotation system. Journal of Agro-Environment Science*,* **35**, 195-204 (in Chinese with English abstract).

Zhang Y (2009) Greenhouse gas emission and carbon balance of a double cropping system in North China Plain. Master thesis, China Agricultural University. (in Chinese with English abstract).

Zhang Y, Liu J, Mu Y, Pei S, Lun X, Chai F (2011) Emissions of nitrous oxide, nitrogen oxides and ammonia from a maize field in the North China Plain. Atmospheric Environment*,* **45**, 2956-2961.

Zhang Y, Liu J, Mu Y, Xu Z, Pei S, Lun X, Zhang Y (2012) Nitrous oxide emissions from a maize field during two consecutive growing seasons in the North China Plain. Journal of Environmental Sciences*,* **24**, 160-168.

Zhang ZX (2006) Effects of urban waste compost and compound fertilizer on nitrous oxide emission from agricultural soils. Master thesis, Capital Normal University. (in Chinese with English abstract).

Zhang ZX, Hua L, Yi XX, Xu ZJ, He TT (2006) Influence of urban waste compost and compound fertilizer on emission of nitrous oxide from agricultural soils. Journal of Agro-Environment Science*,* **25**, 1371-1374 (in Chinese with English abstract).

Zhao JB (2008) Effects of conservation tillage on soil environmental factors and emission of greenhouse gas in the field. PhD thesis, Shandong Agricultural University. (in Chinese with English abstract).

Zhao JB, Chi SJ, Ning TY *et al.* (2008a) Study of N_2_O emissioin and its affecting factors in wheat fields of conservation tillage. Journal of Soil and Water Conservation*,* **22**, 196-200 (in Chinese with English abstract).

Zhao JB, Li ZJ, Chi SJ *et al.* (2008b) CH_4_ absorption and its affecting factors in a wheat field with conservation tillage. Chinese Journal of Applied Ecology*,* **19**, 2490-2496 (in Chinese with English abstract).

Zhou Y, Zhang Y, Tian D, Mu Y (2016) Impact of dicyandiamide on emissions of nitrous oxide, nitric oxide and ammonia from agricultural field in the North China Plain. Journal of Environmental Sciences*,* **40**, 20-27.
